# Adoptive cell therapy with tumor-infiltrating lymphocytes in combination with nivolumab in patients with advanced melanoma

**DOI:** 10.1016/j.iotech.2024.100728

**Published:** 2024-08-22

**Authors:** D. König, B. Kasenda, M. Sandholzer, A. Chirindel, A. Zingg, R. Ritschard, H. Thut, K. Glatz, E.A. Kappos, D. Schaefer, C. Kettelhack, J. Passweg, K. Baur, A. Holbro, A. Buser, D. Lardinois, L.T. Jeker, N. Khanna, F. Stenner, M.S. Matter, N. Rodrigues Mantuano, M. Binder, A. Zippelius, H. Läubli

**Affiliations:** 1 Department of Biomedicine, University of Basel, Basel, Switzerland; 2 Division of Medical Oncology, University Hospital Basel, Basel, Switzerland; 3 Innovation Focus Cell Therapies, University Hospital Basel, Basel, Switzerland; 4 Department of Radiology and Nuclear Medicine, University Hospital Basel, Basel, Switzerland; 5 Institute of Pathology and Medical Genetics, University Hospital Basel, Basel, Switzerland; 6 Department of Plastic, Reconstructive, Aesthetic and Hand Surgery, University Hospital Basel, Basel, Switzerland; 7 Visceral Surgery, Clarunis University Center for Gastrointestinal and Liver Disease, University Hospital Basel and St. Claraspital Basel, Basel, Switzerland; 8 Division of Hematology, University Hospital Basel, Basel, Switzerland; 9 Blood Donation Center, Basel, Switzerland; 10 Department of Thoracic Surgery, University Hospital Basel, Basel, Switzerland; 11 Division of Transplantation Immunology and Nephrology, University Hospital Basel, Basel, Switzerland; 12 Division of Infectious Diseases and Hospital Epidemiology, University Hospital Basel, Basel, Switzerland

**Keywords:** melanoma, adoptive cell therapy, tumor-infiltrating lymphocytes, nivolumab, phase I trial

## Abstract

**Background:**

Adoptive cell therapy (ACT) with tumor-infiltrating lymphocytes (TIL) is a personalized immunotherapy. The efficacy of TIL-ACT has been demonstrated prospectively in patients with advanced melanoma but is not limited to melanoma patients. Many patients are refractory to TIL-ACT, however, or their cancer becomes resistant. Combining anti-programmed cell death protein 1 (anti-PD-1) with TIL-ACT to antagonize the immunosuppressive tumor microenvironment may synergize to enhance the antitumor potential.

**Material and methods:**

We set up the *BaseTIL* trial (NCT04165967), a single-center investigator-initiated phase I trial, to test feasibility and safety of TIL-ACT followed by PD-1 blockade in patients with advanced cutaneous melanoma with disease progression after at least one line of anti-PD-1. TIL-ACT included tumor collection, *ex vivo* TIL expansion, lymphodepletion with cyclophosphamide and fludarabine, TIL transfer, and *in vivo* TIL stimulation with interleukin 2 (125 000 IU/kg, 10 days). TIL-ACT was followed by nivolumab treatment for a maximum of 2 years. Nine patients were planned for inclusion.

**Results:**

Between 2020 and 2022, we enrolled 11 patients and 9 underwent a TIL transfer (median transfused cell number: 66.25 × 10^9^). Two patients did not start lymphodepletion. Nine patients received at least 1 dose of interleukin 2 (median number: 10; range, 1-10), seven started nivolumab (median number: 5; range, 2-23). All patients had hematologic adverse events (AEs). Most common non-hematologic AEs were fever and cytokine release syndrome. No nivolumab-associated AEs of ≥ grade 2 occurred. The objective response rate to TIL-ACT was 22% (2/9, 2 partial remission).

**Conclusions:**

TIL-ACT with nivolumab is feasible and safe. Larger trials are needed to further determine the efficacy of this combination.

## Highlights

• Adoptive cell therapy with tumor-infiltrating lymphocytes and nivolumab is feasible.
• No new safety signals emerged from the combination of TIL-ACT and nivolumab.
• Tumor regression occurred in most patients, but no durable responses.

## Introduction

Adoptive cell therapy (ACT) with tumor-infiltrating lymphocytes (TIL) is a personalized immunotherapy that has been pioneered by Steven A. Rosenberg and colleagues at the National Cancer Institute (NCI).^1^, ^2^, ^3^ TIL-ACT is based on the infusion of autologous CD4+ and CD8+ T lymphocytes that have been collected from tumor material and expanded *ex vivo* in the presence of interleukin 2 (IL-2). Preconditioning with lymphodepletion, most often with non-myeloablative chemotherapy, is an integral part of current TIL-ACT protocols, as well as *in vivo* TIL activation with IL-2 following TIL transfer. Different clinical trials have shown significant response rates in patients with advanced melanoma depending on the stage and patient selection, even after progression on immune checkpoint inhibitors (ICI).^4^^,^^5^ Recently, a randomized phase III trial has demonstrated superior efficacy of TIL-ACT compared with ipilimumab treatment.^6^ In this trial, TIL therapy achieved a 50% reduction in the risk of progression or death compared with ipilimumab in patients with treatment-refractory melanoma. The overall response rate (ORR) was 49% with the TIL therapy compared with 21% for ipilimumab. Activity of TIL-ACT has also been shown in other tumor entities.^7^, ^8^, ^9^

Many patients are still refractory to TIL-ACT, however, or their cancer becomes resistant over time. Long-term responses to TIL-ACT seem to be restricted to patients with complete remissions.^5^^,^^10^ Combining anti-programmed cell death protein 1 (anti-PD-1) inhibitors with TIL-ACT to antagonize the immunosuppressive tumor microenvironment may be a promising strategy to enhance the antitumor potential. The rationale for incorporating a PD-1 inhibitor after TIL-ACT is substantiated by increased PD-L1 expression among tumor-reactive T cells following TIL therapy.^11^

We designed the *BaseTIL* trial (CA209-7H9, NCT04165967), a single-center investigator-initiated phase I trial at the University Hospital Basel, Basel, Switzerland, to investigate feasibility and safety of TIL-ACT followed by PD-1 blockade with nivolumab in patients with advanced melanoma.

## Material and methods

### Patient population

Patients were eligible if they were ≥18 years of age and had histologically confirmed, unresectable stage III or IV melanoma with disease progression after at least one anti-PD-1-based treatment line and additionally: a BRAF inhibitor in patients with BRAF V600 mutation; an accessible metastasis for tumor collection; measurable disease according to Response Evaluation Criteria in Solid Tumors (RECIST), version 1.1; and Eastern Cooperative Oncology Group (ECOG) performance-status score of 0 or 1 (on a 5-point scale, with higher scores indicating greater disability). Key exclusion criteria were non-cutaneous melanoma and active, untreated brain or leptomeningeal metastases.

### Trial design

**Figure 1:**
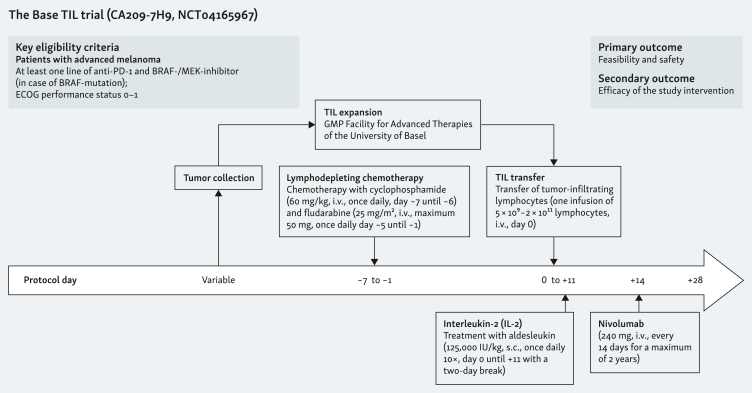
**Study design.** The *BaseTIL* trial a single-center investigator-initiated phase I trial to test feasibility and safety of TIL-ACT followed by PD-1 blockade in patients with advanced cutaneous melanoma. ACT, adoptive cell therapy; ECOG, Eastern Cooperative Oncology Group; IL-2, interleukin 2; TIL, tumor-infiltrating lymphocytes.

In this investigator-initiated phase I trial, patients underwent surgical excision of a metastasis for the generation of the TIL product (Figure 1). Nine patients were planned for TIL treatment in the *BaseTIL* trial. TIL production was carried out at the GMP Facility for Advanced Therapies of the University Hospital of Basel (Supplementary Figure S1 and Supplementary Table S1, available at https://doi.org/10.1016/j.iotech.2024.100728). If generation of the TIL product proceeded as intended, patients started lymphodepleting chemotherapy (LD) intravenously (i.v.) with cyclophosphamide at a dose of 60 mg/kg for day −7 and −6, and fludarabine daily at a dose of 25 mg/m^2^ (maximum of 50 mg) for days −5 to −1. Patients received the TIL infusion on day 0, followed by daily subcutaneous administration of IL-2 (aldesleukin) at a dose of 125 000 IU/kg for 10 days, with a 2-day break after the fifth dose. On day 14, patients started i.v. nivolumab at a dose of 240 mg and were thereafter treated with the same dose every 2 weeks. Treatment with nivolumab continued until the occurrence of disease progression or a maximum duration of 2 years. Patients received support with granulocyte colony-stimulating factor (G-CSF) after TIL infusion and further supportive treatment during TIL-ACT as needed. Prophylaxis for herpes simplex virus and pneumocystis was given after TIL-ACT for the duration of 3 and 6 months, respectively.

### Endpoints and assessments

The primary endpoint was safety of the study intervention by assessment of adverse events (AEs). AEs were graded according to the National Cancer Institute Common Terminology Criteria for Adverse Events, version 4.0. A data and safety monitoring committee (DSMB) met after TIL transfer in three patients. Key secondary endpoints were the assessment of the ORR, progression-free survival (PFS), and overall survival (OS). Tumor response was assessed by the investigators according to the RECIST version 1.1 at 1 month after TIL transfer and then every 3 months. Imaging assessment included computed tomography (CT) scans, [^18^F]2-fluoro-2-deoxy-D-glucose-positron emission tomography (FDG-PET)/CT scans, and magnetic resonance imaging (MRI) scans of the brain at baseline and during the follow-up. PFS was defined as the time from the date of TIL transfer to the date of objective tumor progression or death due to any cause, whichever occurred first. OS was the interval from TIL transfer until death from any cause.

### Trial oversight

The protocol and amendments were reviewed and approved by the local ethics committee (EKNZ, Basel, Switzerland; EKNZ Nr. 2019-01908) and the Swiss Agency for Therapeutic Products (Swissmedic, Bern, Switzerland). The trial was conducted in accordance with the International Council for Harmonization Good Clinical Practice guidelines and the principles of the Declaration of Helsinki. All patients provided written informed consent. The study was designed by the authors. Drug supply of nivolumab was granted by Bristol Myers Squibb, Steinhausen, Switzerland.

### Statistical analyses

Patients’ characteristics were summarized by median and range for continuous variables and by frequency and proportion for categorical variables.

## Results

### Patient population and treatment

Between 2020 and 2022, 11 patients were enrolled in the *BaseTIL* trial and 9 underwent a TIL transfer. Two patients did not start LD: one patient due to rapid disease progression and one patient due to bacterial contamination of the TIL product due to non-adequate starting material. Among the nine patients who had received the TIL infusion (full analysis set), five patients were male and four female (Table 1). The median age was 61 years (range, 35-66 years). Three patients had a *BRAF* mutation. The median number of prior systemic treatment lines, both in the adjuvant and the metastatic settings, was three (range, one to five). Prior systemic treatments included therapy with a PD-1 inhibitor in all patients, and treatment with a BRAF and MEK inhibitor in all three patients with a *BRAF* mutation as required by the study protocol. One patient underwent surgery of a newly diagnosed hemorrhagic brain metastasis and whole brain radiotherapy before the start of lymphodepletion. All patients had poly-metastatic disease (≥5 metastatic lesions) after tumor collection and most patients (5/9) had visceral organ involvement. The median sum of target lesion diameters before TIL treatment was 4.8 cm (range, 2.3-10.4 cm). Median level of blood lactate dehydrogenase at the start of lymphodepletion was 187 U/l (range, 145-265 U/l).

**Table 1:** Patient and disease characteristics and treatment aspects

Patient and disease characteristics	Full analysis set^a^ (*N* = 9)
Median age at TIL transfer (range), years	61 (35-66)
Gender (female/male), *n*	4/5
*BRAF* mutation^b^, *n* (%)	3 (33)
Prior systemic therapies, *n* (%)	
• Median number of therapies (range)	3 (1-5)
• Anti-PD-1	9 (100)
• Anti-CTLA-4	2 (22)
• Combined anti-PD-1/anti-CTLA-4	6 (67)
• BRAF + MEK inhibitor	3 (33)
• Chemotherapy or interferon treatment	3 (33)
• Experimental treatment	2 (22)
Median target lesion sum of diameters (range), mm	48 (23-104)
Presence of brain metastases^c^ (yes/no), *n*	1/8
Median level of LDH (range), U/l	187 (145-265)

CTLA-4, cytotoxic T lymphocyte-associated antigen-4; LDH, lactate dehydrogenase; PD-1, programmed cell death protein 1; TIL, tumor-infiltrating lymphocytes.

a The full analysis set includes nine patients who underwent TIL transfer. Overall, two patients did not start lymphodepleting chemotherapy: one patient due to rapid disease progression; one patient due to bacterial contamination of the TIL product due to non-adequate starting material.

b *BRAF* mutations: V600E, V600E/D, V600E.

c The presence of uncontrolled brain metastases was an exclusion criterion in the study protocol. One patient was diagnosed with brain metastases shortly after study enrollment and underwent surgery of a hemorrhagic brain metastasis and whole brain radiotherapy before the start of lymphodepleting chemotherapy. Stability of brain metastases was documented before start of lymphodepleting chemotherapy.

The median duration of the pre-rapid expansion protocol was 12 days (range, 10-19 days), and for the rapid expansion protocol the median duration was 16 days (range, 14-16 days) (Supplementary Table S2, available at https://doi.org/10.1016/j.iotech.2024.100728). All nine patients started LD as planned. Eight of nine patients received the planned LD (Table 2). One patient had four of the five intended doses of fludarabine. The median absolute number of transfused CD3+ cells was 66.25 × 10^9^ (range, 50-84 × 10^9^). The composition of T cells in the final infusion product and the number of infused cells of every patient are summarized in Supplementary Table S3, available at https://doi.org/10.1016/j.iotech.2024.100728. All patients received at least one dose of IL-2 (*N* = 5 with 10 doses, *N* = 1 with 9 doses, *N* = 2 with 8 doses). One patient was discontinued from the trial after TIL transfer and one dose of IL-2 due to grade 4 cytokine release syndrome (CRS). Seven patients started nivolumab treatment. The median number of nivolumab cycles was 7 (range, 2-23). Two patients were unable to start nivolumab due to logistical reasons (place of residence outside Switzerland, no possibility of local sourcing of nivolumab within the trial).

**Table 2:** Treatment data

Treatment	Full analysis set^a^ (*N* = 9)
Lymphodepleting chemotherapy, *n*	
• Median number of cyclophosphamide doses (range)	2 (2-2)
• Median number of fludarabine doses (range)	5 (4-5)
Median number of TILs infused (range), *n* (×10^9^)	66.25 (50-84)
Median number of IL-2 doses (range), *n*	10 (1-10)
Median single dose of IL-2 (range), *n* (×10^6^ IU)	10.25 (8.13-12.5)
Median number of nivolumab doses (range), *n*	4 (0-23)

IL-1, interleukin 2; TIL, tumor-infiltrating lymphocytes.

a The full analysis set includes 9 patients who underwent TIL transfer.

### Safety

Median duration of hospitalization for TIL-ACT was 25 days (range, 20-44 days). All patients experienced AEs in the period from start of LD (day −7) until 30 days after TIL transfer (Table 3). Hematologic AEs occurred as expected in all patients and were attributed to LD. Highest-grade events were grade 4 white blood count decrease (9/9 patients), grade 4 neutropenia (9/9 patients), grade 4 lymphopenia (9/9 patients), grade 4 platelet count decrease (7/9 patients), and grade 3 anemia (7/9 patients). The most common non-hematological AEs of grade 2 or higher in the above-mentioned period were fever (7/9) and CRS (5/9). One patient experienced a grade 4 CRS, classified as serious AE, which led to trial discontinuation. This patient experienced progressive signs of an acute respiratory distress syndrome (ARDS) resulting in respiratory failure and need of invasive respiratory support shortly after TIL infusion and the first IL-2 dose. Multi-organ failure occurred with cardiac, gastrointestinal, and neurological involvement. The patient received anti-IL-6 targeted treatment (tocilizumab, siltuximab) and high-dose steroid treatment. She fully recovered from the episode but required prolonged hospitalization. The patient did not proceed with any study treatment and did not receive nivolumab. Among the other four patients with grade 2 CRS, three patients received tocilizumab. All patients received antibiotic treatment during TIL-ACT.

**Table 3:** **Adverse events of grade 2 or higher.** Shown are all adverse events (≥grade 2) (*n*, %) of the highest grade recorded for all TIL-treated patients (*N* = 9) from start of lymphodepletion (day −7) until 30 days after TIL transfer (day +30)

CTCAE preferred term, *n* (%)	Any grade	Grade 2	Grade 3	Grade 4
Non-hematologic adverse events				
Fever/chills	7 (78)	7 (78)	0 (0)	0 (0)
Cytokine release syndrome (CRS)	5 (56)	4 (44)	0 (0)	1 (11)^a^
Hypertension	4 (44)	2 (22)	2 (22)	0 (0)
Pain^b^	3 (33)	2 (22)	1 (11)	0 (0)
Liver enzyme elevation^c^	2 (22)	1 (11)	1 (11)	0 (0)
Skin alterations^d^	2 (22)	1 (11)	1 (11)	0 (0)
Hypotension	2 (22)	2 (22)	0 (0)	0 (0)
Nausea/vomiting	2 (22)	2 (22)	0 (0)	0 (0)
Pre-syncope/syncope	2 (22)	2 (22)	0 (0)	0 (0)
Creatinine increased	1 (11)	0 (0)	1 (11)	0 (0)
Wound infection	1 (11)	0 (0)	1 (11)	0 (0)
Atrial fibrillation	1 (11)	1 (11)	0 (0)	0 (0)
Cystitis noninfective	1 (11)	1 (11)	0 (0)	0 (0)
Fatigue	1 (11)	1 (11)	0 (0)	0 (0)
Headache	1 (11)	1 (11)	0 (0)	0 (0)
Mucositis oral	1 (11)	1 (11)	0 (0)	0 (0)
Peripheral sensory neuropathy	1 (11)	1 (11)	0 (0)	0 (0)
Pleural effusion	1 (11)	1 (11)	0 (0)	0 (0)
Hematologic adverse events				
White blood cell decreased	9 (100)	0 (0)	0 (0)	9 (100)
Lymphocyte count decreased	9 (100)	0 (0)	0 (0)	9 (100)
Neutrophil count decreased	9 (100)	0 (0)	0 (0)	9 (100)
Anemia	9 (100)	2 (22)	7 (78)	0 (0)
Platelet count decreased	9 (100)	0 (0)	2 (22)	7 (78)
Hemolysis	1 (11)	0 (0)	1 (11)	0 (0)

TIL, tumor-infiltrating lymphocytes.

a One patient with a CRS (grade 4) with multi-organ failure (classified as serious adverse event).

b One patient with back pain (grade 3), one patient with bone pain (grade 2), one patient with non-cardiac chest pain (grade 2).

c Elevation of alanine aminotransferase (ALT), aspartate aminotransferase (AST) and/or GGT.

d One patient with maculopapular rash (grade 2), one patient with purpura (grade 3).

No nivolumab-associated AEs of ≥ grade 2 occurred. Hyperthyroidism (grade 1) and adrenal insufficiency (grade 1) was documented in one patient, skin hypopigmentation (vitiligo, grade 1) in another patient.

### Efficacy

**Figure 2:**
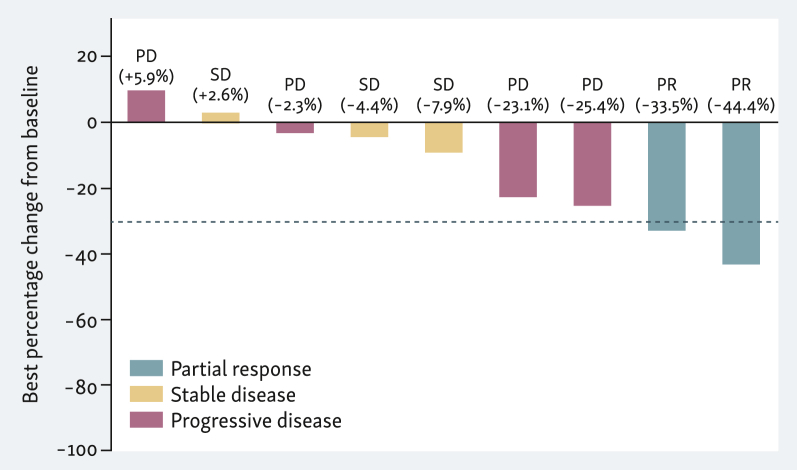
**Best response to the study intervention (waterfall plot).** Shown is the best % change in target lesion sum of diameters from the baseline assessment according to RECIST v1.1 in all nine patients. Censoring occurred at time of progressive disease (PD). The best overall response was partial remission (PR, blue bars) in two patients, stable disease (SD, yellow bars) in three patients, and PD (red bars) in four patients. Shrinkage of target lesions of any magnitude was observed in seven patients. Among the four patients with PD at their first imaging scan, three patients had a shrinkage of target lesions while new lesions were discovered at the same time.

**Figure 3:**
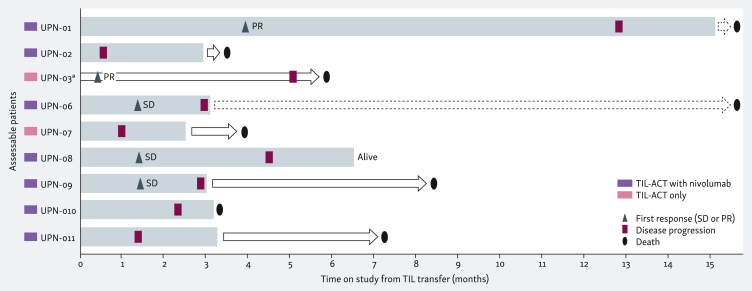
**Time response to the study intervention (swimmer plot).** The bars indicate the time on the study for every patient from the time of TIL transfer until the end-of-study. Radiographic response according to RECIST v1.1 at the first imaging assessment (first response) is described for every patient, including the timepoint of the first imaging assessment. Disease progression is described for every patient, including the time point of disease progression. Best overall response was progressive disease in four patients. The arrow bars indicate the time from the end-of-study until death. Two patients deceased more than 15 months after TIL transfer (dashed arrow bars). ACT, adoptive cell therapy; PR, partial remission; SD, stable disease; TIL, tumor-infiltrating lymphocytes. ∗End-of-study for patient UPN-03 was on the day of TIL transfer. Data cut-off for survival: 15 November 2023.

Of all nine patients, seven patients had a radiographic shrinkage of target lesions of any magnitude at the first imaging scan carried out ∼1 month after TIL-ACT (Figure 2). Three out of these seven patients, however, developed new tumor lesions at their first imaging scan while regression in tumor size was observed in target lesions. Best radiographic response according to RECIST v1.1 was partial remission in two patients, resulting in an ORR of 22%. Stable disease was observed in three patients, while four patients had progressive disease at the first imaging assessment after TIL-ACT. At study end, all patients had experienced disease progression (Figure 3). Median PFS from TIL transfer was 2.2 months. Five patients received subsequent systemic therapies, including the use of combined PD-1/cytotoxic T lymphocyte-associated antigen-4 (CTLA-4) inhibitors in three patients. Median OS from TIL transfer was 7.2 months, with five and three patients alive at 6 and 12 months after TIL transfer, respectively.

## Discussion

The *BaseTIL* trial was one of the first clinical studies that investigated TIL-ACT followed by a PD-1 blocking agent in melanoma patients who experienced disease progression on a previous line of PD-1-based treatment. Because upfront treatment with ICI, most often combined PD-1/CTLA-4 blockade, is standard in patients with advanced melanoma, the question arises as to the optimal therapy for those patients who do not respond to ICI or experience disease progression. TIL therapy has been shown to be an effective treatment option in this disease setting.^6^ Whether the addition of an ICI after TIL therapy is a valid strategy for overcoming resistance to immunotherapy and whether the combination is possibly more effective than TIL therapy alone is currently unclear. The design of the phase I *BaseTIL* trial was aimed at testing feasibility and safety of this combination. With the current study with nine patients including seven patients with the combination of TIL-ACT and nivolumab (two patients did not receive nivolumab for logistical reasons as outlined), we were able to show that feasibility is given. The safety profile of TIL-ACT was in line with that of other clinical trials,^4^ including a pivotal phase III trial.^6^ Toxicities were related mostly to LD and to a lesser extent to IL-2. Importantly, patients in the *BaseTIL* trial received low-dose IL-2 and most patients did not experience dose-limiting toxicities due to IL-2. The rationale for the use of low-dose IL-2 in our trial is based on the assumption of lower toxicity compared with high-dose IL-2, although the results are not clear in this respect.^4^ Importantly, the present study was designed before publication of the results of Rohaan and colleagues,^6^ which established the use of high-dose IL-2 as the standard after TIL transfer. One grade 4 CRS was attributed to the TIL product as has been described in the literature.^12^ Due to the initially predominant pulmonary symptoms (ARDS) and the origin of the expanded TIL product from a pulmonary metastasis, recognition of ‘self-antigen’ expressed by normal lung tissue may be an alternative explanation of the event. Importantly, no higher-grade immune-related AEs occurred during nivolumab treatment. Only seven out of nine patients started nivolumab, however, and the drug exposure was rather short in most patients due to disease progression. In summary, the safety profile of TIL-ACT followed by nivolumab was consistent with the established profiles of the individual treatments, and no new safety signals emerged.

Parameters of efficacy were secondary endpoints in this trial. Interestingly, most patients had some degree of tumor response in the first imaging assessment following TIL-ACT. Apart from one patient who had a long-lasting response of more than 12 months, we did, however, not observe durable responses in our study population. Secondary resistance in our patient with long response was due to selection of a preexisting tumor cell clone due to a mixed histology of epithelioid/desmoplastic melanoma.^13^ The response rate in our study is identical to the ORR of 22% (2/9 patients) in a phase II study of 12 patients with advanced melanoma who received TIL-ACT with a low-dose IL-2 regimen (125 000 IU/kg over 12 days).^14^ Consistent with our results, partial remissions were also not durable in this trial, and no patient achieved a complete remission. Conversely, in the phase I study by Ellebaek and colleagues^15^ at the Center for Cancer Immune Therapy, Denmark, investigating a low-dose IL-2 regimen (2 000 000 IU over 14 days), a sustained complete response to TIL-ACT was seen in two of six patients, indicating that complete remissions can also be achieved with low-dose IL-2. In recent years, however, various studies have increasingly advocated the use of high-dose IL-2. While a meta-analysis of eight TIL studies had already indicated that a higher ORR can be achieved with high-dose (≥720 000 IU/kg) compared with low-dose (<720 000 IU/kg) IL-2 (43% versus 35%), the results of the pivotal phase III study of TIL-ACT versus ipilimumab by Rohaan and colleagues^6^ shifted the standard of care of *in vivo* IL-2 administration towards a high-dose regimen.^4^^,^^6^ From the seven patients in our study who received nivolumab following TIL-ACT, no conclusions can be drawn about the efficacy of this combination. Differences in response rate and duration of response might also be related to the patient selection. Our trial included a population of heavily pre-treated patients with high tumor burden.

The limitation of this trial is the small number of the target population and the heterogeneity of the included patients in terms of previous treatments and treatment lines. Thus, our results contribute to the question of feasibility and safety of the combination of TIL-ACT and PD-1-based treatment, but conclusions about the effectiveness of this intervention are only preliminary. Other clinical trials have investigated TIL therapy in combination with PD-1 blockade.^9^^,^^16^ Both trials concluded in line with our findings that the addition of ICI to TIL-ACT was safe and feasible.

In conclusion, we demonstrated feasibility and safety of TIL-ACT with nivolumab. Larger trials are needed to further determine the efficacy of TIL-ACT with ICI, and possibly explore new combination approaches.
