# Supplementary material for: Adoptive cell therapy with tumor-infiltrating lymphocytes in combination with nivolumab in patients with advanced melanoma
Source: Immunooncol Technol. 2024 Aug 22;24:100728. doi: 10.1016/j.iotech.2024.100728 (PMC11725098; doi:10.1016/j.iotech.2024.100728)
Supplement: Supplementary Material [file mmc1.docx]

*August 12, 2024*

***Supplementary Material***

**Adoptive cell therapy with tumor-infiltrating lymphocytes in combination with nivolumab in patients with advanced melanoma**

**Figure S1. TIL generation at the GMP Facility for Advanced Therapies at the University Hospital Basel, Switzerland for the *BaseTIL* trial.**

In brief, TILs were recruited by IL-2 stimulation from one or multiple resected tumor specimen(s) and were expanded *in vitro* in presence of IL-2, allogenic feeder cells and OKT-3 anti CD3 antibody activation in two phases (pre-rapid expansion protocol, preREP phase; and rapid expansion protocol, REP, phases). The expansion of autologous TIL cells was performed in a clean room environment in a closed system in level A, B and C conditions.

In detail, the starting material for TIL generation was procured entailing one or multiple tumor piece(s) excised from a primary or metastatic tumor lesion. The starting material was cut into small fragments and seeded in 24-well plates for immune cell outgrowth and activation in complete medium with IL-2 cytokine for 3 to 5 days. Subsequently, static cell culture took place for up to day 21 of preREP (5-21 days pre-REP phase; intermediate product). Following preREP, cells (intermediate product) were either directly inoculated for REP manufacture or frozen at this step and stored at -80°C allowing for dynamic patient availability.

For the REP expansion (active substance) TIL cells were thawed and rested for 2 days with IL-2 if cells were frozen. After resting (if frozen) or after direct inoculation the cells were further stimulated with IL-2, anti-CD3 antibody OKT-3 and irradiated allogeneic feeder cells for 7 days in static conditions; and 7 days in a rocking bioreactor without human serum and antibiotics.

After harvest, volume reduction and final formulation in NaCl 0.9% and 2.5% HSA was performed, resulting in the final drug product ready for patient infusion.

**Table S1: The timeline of TIL generation at the GMP Facility for Advanced Therapies at the University Hospital Basel, Switzerland for the *BaseTIL* trial.**

| **Timeline** | **Action** |
| --- | --- |
| Day 0 | Seeding tumor fragments |
| Day 1 – 14 (min. 5; max. 21 days) | Outgrowth, activation and TIL culture |
| Day 7, 10, 14, 17 or 21 | Freezing of TIL (if applicable) |
| Day -2 – 0 of REP | Thawing of TIL (if applicable) |
| Day 0 of REP | Initiation of static REP |
| Day 7 of REP | Medium change and transfer to cell reactor |
| Day 7 – 14 of REP | Feed and perfuse in cell reactor |
| Day 14 REP | Cell harvest for formulation |

**Table S2. Duration of preREP, REP and fold expansion per patient.**

| **Patient** | **Duration of PreREP (days)** | **Duration of REP (days)** | **Fold expansion** |
| --- | --- | --- | --- |
| UPN-01 | 14 | 16 | 4368x |
| UPN-02 | 10 | 16 | 2000x |
| UPN-03 | 10 | 16 | 2800x |
| UPN-06 | 12 | 14 | 2280x |
| UPN-07 | 14 | 16 | 2070x |
| UPN-08 | 10 | 16 | 2661x |
| UPN-09 | 12 | 14 | 2730x |
| UPN-10 | 19 | 14 | 3360x |
| UPN-11 | 17 | 16 | 2530x |
| **Median** | 12 | 16 | 2661x |

**Table S3. Number of infused cells and composition of T cells in the final infusion product per patient.**

| **Patient** | **Infused Cells (x 10^9^)** | **CD45 / CD3+ (Freq. of Parent)** | **CD45 / CD3+ / CD8+ (Freq. of Parent)** | **CD45 / CD3+ / DP CD4+ CD8+ (Freq. of Parent)** | **CD45 / CD3+ / CD4+ (Freq. of Parent)** | **CD45 / CD3+ / DN CD4- CD8- (Freq. of Parent)** | **CD45 / CD3+ CD16_CD56+ (Freq. of Parent)** | **CD45 / CD3+ CD16_CD56 (Freq. of Parent)** | **CD45 / NK CD3- CD16_CD56+ (Freq. of Parent)** |
| --- | --- | --- | --- | --- | --- | --- | --- | --- | --- |
| UPN-01 | 74.25 | 98.60% | 79.90% | 5.25% | 14.10% | 0.80% | 14.80% | 84.90% | 0.02% |
| UPN-02 | 50 | 97.60% | 17.70% | 0.08% | 1.41% | 80.80% | 33.30% | 66.60% | 0.09% |
| UPN-03 | 70 | 99.50% | 91.20% | 1.24% | 6.80% | 0.76% | 19.00% | 80.70% | 0.14% |
| UPN-06 | 57 | 98.40% | 47.50% | 7.48% | 43.40% | 1.68% | 28.20% | 70.80% | 0.33% |
| UPN-07 | 51.75 | 97.80% | 79.80% | 6.33% | 11.50% | 2.45% | 22.90% | 75.30% | 0.41% |
| UPN-08 | 66.25 | 99.00% | 82.50% | 3.91% | 7.63% | 5.93% | 26.80% | 72.20% | 0.24% |
| UPN-09 | 68.25 | 98.80% | 94.00% | 1.62% | 3.29% | 1.10% | 32.40% | 66.60% | 0.90% |
| UPN-10 | 84 | 99.00% | 98.00% | 0.39% | 1.28% | 0.33% | 75.90% | 23.50% | 0.40% |
| UPN-11 | 63.25 | 97.40% | 51.60% | 4.30% | 42.90% | 1.25% | 21.50% | 77.40% | 0.21% |
| **Mean** |  | 98.50% | 71.40% | 3.40% | 14.70% | 10.60% | 30.50% | 68.70% | 0.30% |
